# Biofilms in Wounds: New Advances in Therapy and in Healing Management

**DOI:** 10.3390/biomedicines9020193

**Published:** 2021-02-16

**Authors:** Célia F. Rodrigues, Karishma S. Kaushik, Caitlin Light

**Affiliations:** 1 LEPABE—Department of Chemical Engineering, Faculty of Engineering, University of Porto, 4200-465 Porto, Portugal; 2 Institute of Bioinformatics and Biotechnology, Savitribai Phule Pune University, Pune 411007, India; karishmaskaushik@gmail.com; 3 First-Year Research Immersion Program, Binghamton Biofilm Research Center, Binghamton University, Binghamton, NY 13904, USA; clight@binghamton.edu

Biofilms are the major way of life for both bacteria and fungi. These microbial communities are implicated in the development of many infection states, which are highly recalcitrant to antimicrobial treatments [1]. In fact, the majority of non-healing wounds have associated biofilm infection, which contribute to the high global cost of chronic wound management. Biofilm infected, non-healing have a low-grade and persistent inflammatory response, which leads to impaired epithelialization and granulation tissue formation, and reduced susceptibility to antimicrobial agents. Additionally, a compromised host defense severely delays the healing of wounds in patients, contributing to persistent infection [2].

Therefore, developing approaches to mitigate biofilms in wounds is, indeed, a critical element of effective wound care. For this, strategies to manage wound biofilms and encourage progression of wound healing are urgently needed.

We thank all the authors who published their relevant work in this Special Issue—Biofilms in Wounds: New Advances in Therapy and in Healing Management. In their research paper, Haq et al. [3] show that polymeric films and hydrogels can be useful for the control of *Staphylococcus aureus* infections and disinfection of skin wounds. The authors used these platforms, and thymoquinone as an antimicrobial compound, which was observed to significantly reduce in vitro and in vivo infection. This work revealed that this approach has great potential in treating and managing wound infections. Next, Trane and colleagues [4] addressed the possibility of developing polyester-coated bandages to reduce in vivo biofilm formation in wounds. Dressing materials are known to be easily colonized by bacteria, promoting wound re-infection. Given this, the design and material of bandages could serve as an approach to reduce the development of biofilms in wounds. These authors used an organo-selenium (OS)-coated polyester dressing and studied its effectiveness on the growth inhibition of common wound pathogens, such as *Staphylococcus aureus*, *Stenotrophomonas maltophilia*, *Enterococcus faecalis*, *Staphylococcus epidermidis*, and *Pseudomonas aeruginosa*. This novel bandage was able to reduce 100% of the bacteria on the material of the OS-coated wound dressing and in the underlying tissue. These results indicate that this bandage might have important clinical applications. In their review paper, Kadam et al. [5] carefully examine the current advances in non-conventional antimicrobial approaches for chronic wound biofilms. The work is is organized by the review of three main strategies for combating biofilm wound infections: (1) non-conventional approaches that directly kill or inhibit microorganisms in chronic wound biofilms, using mechanisms or delivery strategies divergent from conventional antibiotics; (2) approaches that change the biological, chemical or biophysical factors in the chronic wound microenvironment (disruption and removal of biofilms); (3) and methods that affect both biofilm bacteria and microenvironment factors. Finally, Bahamondez-Canas et al. [6] published an up-to-date status of in vitro models and susceptibility testing assays for wound biofilms, focusing on *Pseudomonas aeruginosa* and *Staphylococcus aureus* (single and mixed biofilms), mammalian cells, simulant fluids, and tissue explants in order to mimic the physiological states of an infection site. Together, this Special Issue provides the scientific community with current, novel and relevant advances related to the therapeutic challenges of biofilms and their relationship with persistent infections.
